# Clinical Results of Proton Beam Therapy for Esophageal Cancer: Multicenter Retrospective Study in Japan

**DOI:** 10.3390/cancers11070993

**Published:** 2019-07-16

**Authors:** Takashi Ono, Hitoshi Wada, Hitoshi Ishikawa, Hiroyasu Tamamura, Sunao Tokumaru

**Affiliations:** 1 Department of Radiation Oncology, QST Hospital, 4-9-1 Anagawa, Inage-ku, Chiba-shi, Chiba 263-8555, Japan; 2 Department of Radiation Oncology, Southern Tohoku Proton Therapy Center 7-172, Yatsuyamada, Koriyama, Fukushima 963-8052, Japan; 3 Department of Radiation Oncology and Proton Medical Research Center, University of Tsukuba, 2-1-1, Amakubo, Tsukuba, Ibaraki 305-8576, Japan; 4 Department of Radiation Oncology, Proton Therapy Center, FukuiPrefectural Hospital, 2-8-1, Yotsui, Fukui-shi, Fukui-ken 910-8526, Japan; 5 Department of Radiology, Hyogo Ion Beam Medical Center, 1-2-1, Koto, Shingu-cho, Tatsuno City, Hyogo 679-5165, Japan

**Keywords:** proton therapy, esophageal neoplasms, pericardial effusion

## Abstract

There are few reports about the clinical results of proton beam therapy for esophageal cancer in a large population. The purpose of this study was to evaluate the clinical results of proton beam therapy for esophageal cancer in a large population using a multicentered database. Between January 2009 and December 2013, patients newly diagnosed with esophageal cancer and who had received proton beam therapy were retrospectively recruited from a database of four proton beam therapy centers in Japan. Two hundred and two patients (including 90 inoperable patients) fulfilled the inclusion criteria, and 100 patients (49.5%) had stage III/IV cancer (Union for International Cancer Control 8th). The 3-year and 5-year overall survival rate was 66.7% and 56.3%, respectively. The five-year local control rate was 64.4%. There were two patients with grade three pericardial effusion (1%) and a patient with grade three pneumonia (0.5%). No grade 4 or higher cardiopulmonary toxicities were observed (Common Terminology Criteria for Adverse Events version 4.0). This study suggests that proton beam therapy for esophageal cancer was not inferior in efficacy and had lower rates of toxicities in comparison to photon radiotherapy. Therefore, proton beam therapy can serve as an alternate treatment for patients with esophageal cancer.

## 1. Introduction

Esophageal cancer is the sixth leading cause of cancer-related deaths and the seventh most common cancer worldwide [1]. The incidence of esophageal cancer is highest in East Asia, and it is the seventh most common cause of cancer-related deaths in Japan [2]. With an aging society, the number of older patients diagnosed with esophageal cancer is increasing. In Japan, in 2010, 39.1% of patients with esophageal cancer were aged ≥70 years, and squamous cell carcinoma accounted for 90.5% of all esophageal cancer cases [3].

For operable esophageal cancer, surgery remains the primary choice of treatment in Japan, with 61.9% of individuals undergoing such treatment [3]. After conducting a randomized control study comparing preoperative versus postoperative chemotherapy, the Japan Clinical Oncology Group (JCOG) 9907 trial has shown that preoperative chemotherapy as an adjunct to surgery was the standard choice of treatment for operable esophageal cancer in Japan [4].

However, subsequent reports from the Radiation Therapy Oncology Group [5,6] have shown that chemoradiotherapy (CRT) has become an important alternative for the treatment of esophageal cancers to keep the esophagus intact. Moreover, CRT may be an important alternative for inoperable esophageal cancer. Clinical trials of the efficacy and toxicity of CRT, which is used for the treatment of esophageal cancer, have been conducted in Japan [7,8,9], and 24.0% of patients with esophageal cancer received CRT as first-line treatment in 2010 [3].

Compared to conventional photon radiotherapy or intensity-modulated radiotherapy (IMRT), proton beam therapy (PBT), a new radiotherapy technology, delivers concentrated doses of proton beams to the target tissues with minimal harm to surrounding normal tissues [10,11,12,13,14]. Such therapies are safer alternatives with higher efficacy. Between 1954 and 2013, more than 120,000 patients have been treated with particle therapy worldwide and approximately 88% have received PBT [15]. In Japan, >2500 patients received PBT in 2013 [16].

The use of PBT with or without chemotherapy for the treatment of esophageal cancer is increasing [17,18,19,20,21,22,23,24]. However, data about its safety and efficacy are limited. In this multicenter study, we retrospectively evaluated the efficacy and safety of PBT.

## 2. Results

### 2.1. Patients

A total of 202 patients fulfilled the inclusion criteria. The baseline characteristics of the cohort, which included 90 (44.1%) patients with inoperable cancer (due to esophageal cancer, comorbidities, or other conditions), are summarized in Table 1. The study population comprised 167 men and 35 women with a median age of 69 (range: 36–90) years. The median follow-up time was 47 (range: 2–112) months. Lymph node metastasis was observed in 113 (56.0%) patients, and 100 (49.5%) patients had stage III and IV cancer. The median total dose of the biological effective dose (BED) 10 was 87.2 Gy (relative biological effectiveness (RBE); range: 67.2–96.1 Gy (RBE)). Differences were observed in the treatment methods in the four PBT centers. The elective nodal irradiation (ENI) field was based on the location of the primary esophageal cancer and treatment decisions of the institutions. That is, ENI included sites from the bilateral supraclavicular lymph node to the celiac artery, from the aortic arch to the perigastric area, or from the hyoid bone to the carina (which included the bilateral supraclavicular lymph node). The gross tumor volume (GTV) indicated primary tumor and lymph node metastasis based on endoscopic and imaging findings. The clinical target volume (CTV) was defined as 2–5-cm margins for both cranial and caudal edges and 0.5–2-cm margins from other directions. The planning target volume was defined as CTV plus 0.5–1 cm. The daily X-ray imaging finding was used for positioning with bone and clips, which were placed before treatment via endoscopy in all four PBT centers. If possible, 2–4 cycles of chemotherapy were performed concurrently and after PBT in four PBT centers. The most commonly used chemotherapy regimen was cisplatin and 5-fluorouracil (cisplatin 70 mg/m^2^ on day 1 and 5-fluorouracil 700 mg/m^2^/24 h on days 1–4). The second most commonly used regimen was nedaplatin and 5-fluorouracil (nedaplatin 130 mg/m^2^ on day 6 and 5-fluorouracil 700 mg/m^2^/24 h on days 1–5).

### 2.2. Survival

**Figure 1:**
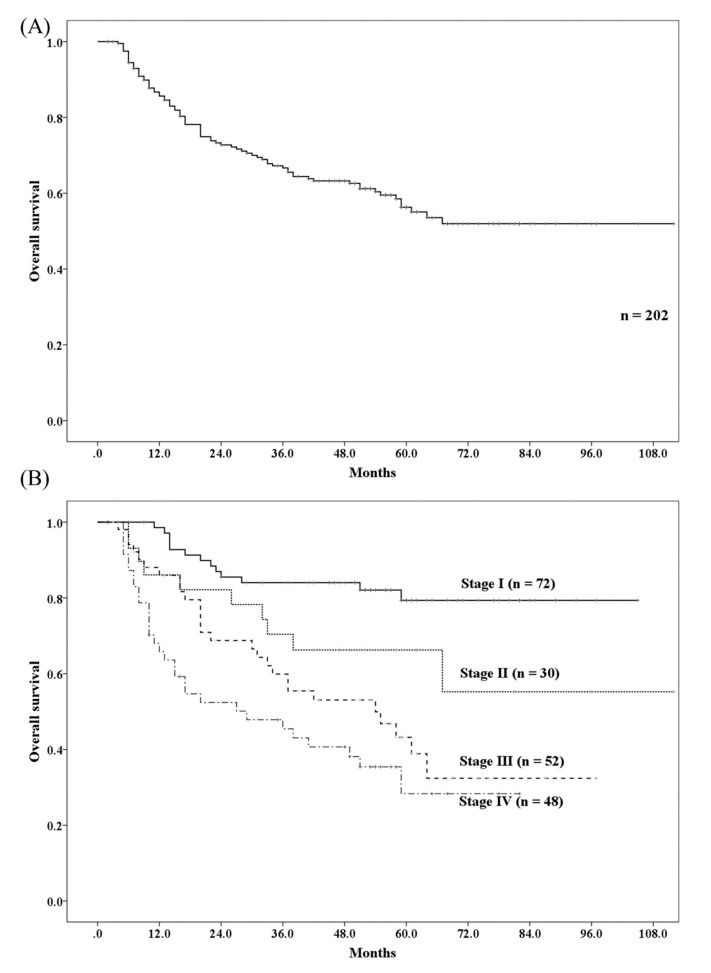
Overall survival rate of patients with esophageal cancer received proton beam therapy. (**A**) Overall survival rate for all patients. (**B**) Overall survival rate for each stage.

A total of 81 patients died of whom, 60 died from esophageal cancer and 21 from other causes (including 4 patients with a new cancer occurrence after treatment for esophageal cancer). The 3- and 5-year overall survival (OS) rates were 66.7% (95% confidence interval (CI): 60.0–73.4%) and 56.3% (95% CI: 48.7–63.9%), respectively (Figure 1A). The 5-year OS rate based on cancer stages I, II, III, and IV were 79.3%, 66.3%, 43.2%, and 28.3%, respectively (Figure 1B). Age ≥ 70 years, performance status 1–3, inoperable tumor status, T3–4, N1–3, stages III and IV, and supraclavicular lymph node metastasis were found to be significant factors for OS in the univariate analysis. However, on subsequent multivariate analyses, only performance status 1–3, inoperable tumor status, and T3–4 were found to be significant factors for OS (Table 2).

### 2.3. Failure Patterns

**Figure 2:**
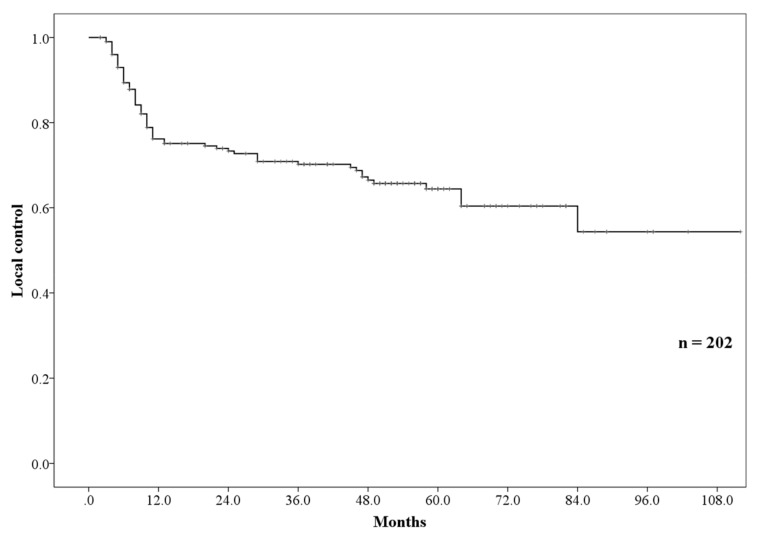
Local control rate.

Ninety-eight patients presented with recurrence. Fifteen patients had lymph node recurrence, including in- and out-field recurrence; 17, distant metastases; and 66, local recurrence. The 3- and 5-year local control (LC) rates were 70.2 (95% CI: 63.5–76.9%) and 64.4% (95% CI: 57.0–71.8%), respectively (Figure 2). Inoperable tumor status, T3–4, N1–3, and stages III and IV were the significant factors for LC in the univariate analysis. However, in subsequent multivariate analyses, inoperable tumor status was found to be the only significant factor for LC (Table 3).

### 2.4. Toxicities

No grade 4 or 5 toxicities were recorded after treatment (Table 4). Two patients who underwent pericardiocentesis (grade 3) received ENI using photon radiotherapy from the bilateral supraclavicular lymph node region to the celiac artery lymph node region. Eight patients had an esophageal fistula with a locally advanced esophageal cancer (T4 category (*n* = 6) and T3 category (*n* = 2)). One patient who presented with pneumonia required oxygenation (grade 3).

## 3. Discussion

We evaluated the safety and efficacy of PBT for the treatment of esophageal cancer using multicenter data in Japan. To the best of our knowledge, our study had the largest cohort of patients treated with PBT for esophageal cancer worldwide.

Previous clinical trials have shown that the 5-year OS for locally advanced esophageal cancer is 25–37% [5,8,9]. The OS of patients with stage III cancer in the present study was slightly higher than that in previous trials. In Japan, patients with stage IV esophageal cancer also receive radical treatment if they did not present with distant organ metastasis. Jingu et al. have reported a 4-year OS of 24.4% in patients with stage IV esophageal cancer who underwent CRT [24]. The OS of the present study was higher than that of the study of Jingu et al. Although the OS may decrease with further follow-up, these results indicate that PBT is not inferior to photon radiotherapy in terms of efficacy for the treatment of esophageal cancer. No prospective studies have directly compared PBT and photon radiotherapy. Recently, Xi et al. have conducted a retrospective study on a cohort with stage I–III esophageal cancer treated with either PBT (*n* = 132) or IMRT (*n* = 211) [25]. Results showed that patients who received PBT had a significantly higher 5-year OS rate than those who did not (41.6% vs. 31.6%). Further, in the multivariate analysis, PBT was a significant factor for OS. Similarly, our findings showed an improvement in OS in patients with esophageal cancer receiving PBT. However, in the study by Xi et al., the causes for the improvement in OS were not identified.

A higher rate for localized recurrence is a major problem in definitive radiotherapy for esophageal cancer and is observed in 34–46% of patients with locally advanced esophageal cancer in a previous study [5,8]. The rate of LC observed in our study was slightly higher than or equal to that of previous studies even after including patients with stage IV esophageal cancer, which could be attributed to the higher total dose used in this study. Some reports have shown a correlation between LC and increased dose [26,27,28]. Suh et al. have reported that patients receiving CRT and a total dose > 60 Gy had a better LC [26]. In a separate study, Zhang et al. have shown that significantly higher rates of LC and OS were observed in patients receiving a dose > 51 Gy [27]. A meta-analysis on preoperative CRT for the treatment of esophageal cancer has indicated that a higher dose was correlated to a higher pathological complete response [28]. Local recurrence of esophageal cancer can decrease a patient’s quality of life; therefore, an increase in dose using PBT may be useful in improving LC particularly in patients with inoperable esophageal cancer.

In patients with esophageal cancer, heart toxicity is a significant problem after radiotherapy. In previous clinical trials, 6.9–16% of patients presented with ≥grade 3 had pericardial effusions after treatment [8,9]. The incidence rate of pericardial effusion in this study was lower than that in previous studies, which may be due to the fact that the irradiated dose in the heart was reduced using PBT. In fact, both patients who had grade 3 pericardial effusions also received ENI using photon radiotherapy. Makishima et al. and Hirano et al. have reported that PBT can significantly reduce the irradiated dose in the heart [13,14]. Further, Makishima et al. have investigated the actual incidence rate of heart toxicities and reported that a lower incidence rate of ≥grade 2 heart toxicities was observed in patients who received PBT (4% vs. 53%) [13]. These results indicated that PBT can reduce the incidence of pericardial effusion and the need for treatment of adverse events after CRT. A large population-based study of heart disease-related mortality using surveillance, epidemiology, and outcome results has found that definitive radiotherapy for esophageal cancer and age were the predictive factors of heart disease-related mortality [29]. Although age was not found to be a significant factor for OS in our study, the previous finding showed that definitive radiotherapy for the treatment of esophageal cancer increased heart disease-related mortality and decreased OS in older patients. Conversely, Hayashi et al. have reported that the dose-volume histogram parameters of the heart are strong independent predictive factors of pericardial effusion [30]. Therefore, decreasing the irradiated dose in the heart using PBT may lead to a decrease in the incidence of heart toxicities after definitive radiotherapy, which is beneficial for patients with middle or lower thoracic esophageal cancer near the heart due to heart sparing. In Japan, 81% of patients with esophageal cancer have middle or lower thoracic cancer [3]; therefore, PBT was found to be beneficial in numerous patients. Moreover, because 39.1% of patients with esophageal cancer patients are aged ≥70 years in Japan [3], a decrease in the incidence rate of heart toxicities using PBT is useful.

Pneumonitis is another side effect post CRT in patients with esophageal cancer. Previous studies have reported an occurrence rate of 3.3–11.7% ≥ grade 2 pneumonitis after CRT [8,9]. Asakura et al. reported that a higher amount of irradiated lung volume might lead to a higher occurrence rate of pneumonitis [31]. Tanabe et al., have also reported a correlation between irradiated lung volume and occurrence rate of pneumonitis [32]. These studies have also shown that an increase in the lung volume exposed to a low dose was also associated with an incidence of pneumonitis. With respect to the irradiated volume in PBT, a larger difference in irradiated mean lung volume was found in lower dose areas [13,14]. Our results, in congruence with prior results, suggest that PBT can reduce pneumonitis after definitive radiotherapy.

There were 2 limitations in the present study. First, the study consists most of patients affected by cervical-thoracic esophageal cancer patients, and only one case of cancer arising from the esophageal-gastric junction is included in this series due to the epidemiological characteristics of esophageal cancer in Japan. However, the overall results are not likely to be reproducible in western countries, where the incidence of lower third cancers and adenocarcinomas is more common. In fact, Bollschweiler et al. have reported that squamous cell carcinoma was more sensitive to radiotherapy and chemotherapy than was adenocarcinoma [33]. Second, some differences were observed in terms of treatment in the four PBT centers. However, the large clinical data, which include those of toxicities, are important. Further prospective studies must be conducted to evaluate such findings.

## 4. Materials and Methods

### 4.1. Ethics Statement

This retrospective multicenter study was approved by each ethics committees of all of 4 institutions (Southern Tohoku Proton Therapy Center, 236-4, approved on 5 October 2017; University of Tsukuba, H29-282, approved on 16 February 2018; Fukui Prefectural Hospital, 17-33, approved on 13 November 2017; Hyogo Ion Beam Medical Center, 29-18, approved on 20 October 2017). The study was conducted in accordance with the Declaration of Helsinki (236-4, approved on 5 October 2017).

### 4.2. Patients

Between January 2009 and December 2013, patients newly diagnosed with esophageal cancer and treated with PBT, with or without chemotherapy, were retrospectively recruited from a database of 4 PBT centers in Japan. The diagnosis of esophageal cancer was histologically confirmed for all patients based on a biopsy results obtained prior to each treatment. All patients were assessed for their clinical stage of esophageal cancer (Union for International Cancer Control 8th edition) using endoscopy, esophagram, computed tomography (CT), and positron emission tomography-CT.

The inclusion criteria were as follows: A histologically confirmed diagnosis of squamous cell carcinoma or adenocarcinoma of esophagus, absence of metastases to distant organs, no other sites with uncontrolled cancer within 5 years before this treatment, and no prior treatments for esophageal cancer. Prior photon radiotherapy for ENI, which was the prophylactic lymph node irradiation, was allowed, because PBT fields were not large to cover ENI in all center.

### 4.3. Evaluation of Toxicities

Toxicities associated with the use of PBT were evaluated using the Common Terminology Criteria for Adverse Events version 4.0 (The US National Cancer Institute, Bethesda, MD, USA) [34]. We assessed the incidence of esophageal ulcers, esophageal fistulas, pericardial effusion, pleural effusion, and pneumonitis.

### 4.4. Statistical Analysis

The reported dose of PBT was calculated by multiplying 1.1 considering RBE. Because 1.8–2.2 Gy (RBE) per fraction was used, the effects of PBT on esophageal cancer were comparable with those of BED. An alpha/beta ratio of 10 Gy was used to calculate BED using a linear-quadratic model, which were as follows: BED Gy (RBE) = total dose × (1 + dose per fraction/10). All statistical analyses were carried out using the Statistical Package for the Social Sciences software (version 22, SPSS Inc., Chicago, IL, USA). The overall OS time was defined as the time between the start of treatment and the last follow-up. In this study, the LC includes both LC locations of high dose and marginal areas. The LC time was defined as the time between the start of treatment and the date on which tumor recurrence was found or the last follow-up. A Kaplan–Meier test was used to estimate OS and LC. Univariate analysis was performed via univariate Cox regression analysis. Factors with a *p*-value < 0.1 in the univariate analysis were used in the multivariate Cox regression analysis. All *p*-values were two-sided, and *p* values < 0.05 were considered statistically significant.

## 5. Conclusions

PBT was not inferior to photon radiotherapy in terms of efficacy for the treatment of esophageal cancer, and the use of such treatment resulted in a lower incidence of cardiopulmonary toxicities. Thus, PBT can be used as an alternative treatment in patients with esophageal cancer.

## Figures and Tables

**Table 1:** Patient characteristics.

Characteristics	Patients
Follow up time	
Median (range)	47 (2–112) months
Gender	
Male	167 (83.7%)
Female	35 (17.3%)
Age	
Median (range)	69 (36–90) years
Performance status	
0	126 (62.4%)
1	52 (25.7%)
2	21 (10.4%)
3	3 (1.5%)
T category ^†^	
T1	74 (36.6%)
T2	31 (15.4%)
T3	62 (30.7%)
T4	35 (17.3%)
N category ^†^	
N0	89 (44.0%)
N1	67 (33.2%)
N2	36 (17.8%)
N3	10 (5%)
Stage ^†^	
I	72 (35.6%)
II	30 (14.9%)
III	52 (25.7%)
IV	48 (23.8%)
Tumor location	
Cervical	20 (9.9%)
Thoracic	181 (89.6%)
Abdominal	1 (0.5%)
Histopathology	
Squamous cell carcinoma	195 (96.5%)
Adenocarcinoma	7 (3.5%)
Total dose (BED 10)	
Median (range)	87.2 (67.2–96.1) Gy (RBE)
Elective nodal irradiation	
None	57 (28.2%)
Using proton beam therapy	26 (12.9%)
Using photon radiotherapy	119 (58.9%)
Chemotherapy	
Cisplatin and 5-fluorouracil	92 (45.5%)
Nedaplatin and 5-fluorouracil	48 (23.8%)
Tegafur, Gimeracil, Oteracil Potassium	9 (4.5%)
Docetaxel and Cisplatin and 5-fluorouracil	3 (1.5%)
Cisplatin	1 (0.5%)
None	49 (24.2%)

Abbreviations: BED: Biological effective dose; RBE: Relative biological effectiveness. ^†^ Numbers correspond to the tumor-node-metastasis system of classification (Union for International Cancer Control) 8th.

**Table 2:** Univariate and multivariate analysis for overall survival.

Factor	Patient (*n* = 202)	5 Year OS	Univariate Analysis		Multivariate Analysis	
			HR (95% CI)	*p*-Value	HR (95% CI)	*p*-Value
Age			1.67 (1.07–2.61)	0.023 *	1.25 (0.74–2.12)	0.397
<70	105	63.4%				
≥70	97	48.2%				
Gender			0.77 (0.42–5.18)	0.409	-	-
women	35	67.1%				
men	167	54.0%				
Performance status			2.81 (1.80–4.37)	<0.001 *	2.14 (1.23–3.74)	0.007 *
0	126	66.8%				
1–3	76	38.8%				
Operability			3.33 (2.11–5.28)	<0.001 *	1.69 (1.01–2.84)	0.045 *
operable	112	71.8%				
inoperable	90	35.7%				
T category			4.07 (2.48–6.66)	<0.001 *	2.30 (1.03–5.13)	0.041 *
T1/2	105	76.2%				
T3/4	97	35.7%				
N category			2.89 (1.76–4.76)	<0.001 *	2.06 (0.99–4.30)	0.055
N0	89	73.3%				
N1–3	113	47.8%				
M1 lymph node metastasis			2.15 (1.07–4.32)	0.031 *	1.51 (0.68–3.34)	0.310
no	188	58.4%				
yes	14	30.1%				
Stage			3.43 (2.11–5.58)	<0.001 *	0.95 (0.38–2.38)	0.904
1/2	102	75.4%				
3/4	100	36.8%				
Total dose (BED 10)			0.73 (0.46–1.16)	0.180	-	-
≥87.2 Gy (RBE)	113	52.0%				
<87.2 Gy (RBE)	89	62.0%				
Elective nodal irradiation			1.10 (0.68–1.78)	0.691	-	-
yes	145	57.7%				
no	57	51.8%				
Chemotherapy			1.58 (0.97–2.57)	0.064	1.21 (0.67–2.20)	0.530
yes	153	60.5%				
no	49	40.3%				

Abbreviations: OS: Overall survival; HR: Hazard ratio; CI: Confidential interval; BED: Biological effective dose; RBE: Relative biological effectiveness. * *p*-value < 0.05.

**Table 3:** Univariate and multivariate analysis for local control.

Factor	Patients (*n* = 202)	5 Year LC	Univariate Analysis		Multivariate Analysis	
			HR (95% CI)	*p* Value	HR (95% CI)	*p* Value
Age			1.12 (0.69–1.81)	0.654	-	-
<70	105	65.6%				
≥70	97	63.0%				
Gender			0.70 (0.35–1.41)	0.315	-	-
women	35	75.5%				
men	167	62.0%				
Performance status			1.47 (0.90–2.40)	0.122	-	-
0	126	67.5%				
1–3	76	59.5%				
Operability			2.19 (1.34–3.58)	0.002 *	1.81 (1.09–3.03)	0.023 *
operable	112	72.4%				
inoperable	90	53.0%				
T category			2.29 (1.39–3.58)	0.001 *	2.10 (0.97–4.54)	0.060
T1/2	105	76.9%				
T3/4	97	49.3%				
N category			1.97 (1.18–3.30)	0.010 *	1.79 (0.83–3.82)	0.136
N0	89	75.5%				
N1–3	113	54.9%				
M1 lymph node metastasis			1.00 (0.37–2.76)	0.993	-	-
no	188	64.3%				
yes	14	70.1%				
Stage			1.81 (1.11–2.97)	0.018 *	0.60 (0.23–1.54)	0.285
1/2	102	74.0%				
3/4	100	53.8%				
Total dose (BED 10)			1.48 (0.91–2.39)	0.116	-	-
≥87.2 Gy (RBE)	113	70.7%				
<87.2 Gy (RBE)	89	57.1%				
Elective nodal irradiation			1.18 (0.70–1.99)	0.546	-	-
yes	145	65.9%				
no	57	59.5%				
Chemotherapy			1.34 (0.77–2.33)	0.298	-	-
yes	153	66.8%				
no	49	52.9%				

Abbreviations: LC: Local control; HR: Hazard ratio; CI: Confidential interval; BED: Biological effective dose; RBE: Relative biological effectiveness. * *p*-value < 0.05.

**Table 4:** Toxicities.

Toxicities	Grade 0/1	Grade 2	Grade 3	Grade 4/5
Esophageal ulcer	122 (60.4%)	72 (35.6%)	8 (4.0%)	0
Esophageal fistula	194 (96.0%)	8 (4.0%)	0	0
Pericardial effusion	170 (84.2%)	30 (14.8%)	2 (1.0%)	0
Pleural effusion	191 (94.6%)	11 (5.4%)	0	0
Pneumonitis	199 (98.5%)	2 (1.0%)	1 (0.5%)	0
